# Reliability and validity of proxy-SSPedi and mini-SSPedi in pediatric patients 2-7 years receiving cancer treatments

**DOI:** 10.1186/s12885-022-09814-8

**Published:** 2022-07-04

**Authors:** Deborah Tomlinson, L. Lee Dupuis, Donna L. Johnston, Susan Kuczynski, Serina Patel, Tal Schechter, Emily Vettese, Mark Mairs, George A. Tomlinson, Lillian Sung

**Affiliations:** 1 Program in Child Health Evaluative Sciences, The Hospital for Sick Children, Peter Gilgan Centre for Research and Learning, 686 Bay Street, Toronto, Ontario M5G 0A4 Canada; 2 Department of Pharmacy, The Hospital for Sick Children555 University Avenue, Toronto, Ontario M5G 1X8 Canada; 3 Leslie Dan Faculty of Pharmacy, University of Toronto, Toronto, Ontario M5S 3M2 Canada; 4 Division of Hematology/Oncology, Children’s Hospital of Eastern Ontario, 401 Smyth Road, Ottawa, Ontario K1H 8L1 Canada; 5 Ontario Parents Advocating for Children with Cancer (OPACC), 99 Citation Drive, Toronto, Ontario M2K 1S9 Canada; 6 Division of Haematology/Oncology, Department of Pediatrics, London Health Sciences Centre, 800 Commissioners Road East, London, Ontario N6A 5W9 Canada; 7 Division of Haematology/Oncology, The Hospital for Sick Children, 555 University Avenue, Toronto, Ontario M5G 1X8 Canada; 8 Department of Medicine, Toronto General Hospital, 200 Elizabeth Street, Toronto, Ontario M5G 2C4 Canada

**Keywords:** Symptom screening, Children, Validity, Reliability, Responsiveness, Proxy, Oncology, Hematopoietic stem cell transplantation

## Abstract

**Background:**

Symptom Screening in Pediatrics Tool (SSPedi) was developed for symptom screening by children 8-18 years. Objectives were to evaluate the reliability and validity of proxy-SSPedi and self-report mini-SSPedi for younger children.

**Methods:**

This multi-center study enrolled guardians of children 2-7 years receiving cancer treatments (proxy-SSPedi) and their children 4-7 years (mini-SSPedi). The two populations were: (1) More symptomatic group where children were receiving active cancer treatment and were in hospital or clinic for four consecutive days; and (2) Less symptomatic group where children were receiving maintenance therapy for acute lymphoblastic leukemia or had completed cancer therapy. Proxy-SSPedi or mini-SSPedi were completed with measures of mucositis, nausea, pain, quality of life and overall symptoms. Respondents in the more symptomatic group repeated proxy-SSPedi/mini-SSPedi and a global symptom change scale 3 days later.

**Results:**

There were 402 guardians and 326 children included in the analysis. Test re-test reliability of proxy-SSPedi showed intraclass correlation coefficient (ICC) 0.83 (95% confidence interval (CI) 0.72-0.90). Mean difference in proxy-SSPedi between more and less symptomatic groups was 9.7 (95% CI 8.3-11.1). Proxy-SSPedi was responsive to change and hypothesized relationships between measures were observed. With a priori threshold ≥0.6, inter-rater ICC among all dyads and those 6-7 years were 0.54 (95% CI 0.45-0.62) and 0.62 (95% CI 0.50-0.71) respectively. Among participating children, other hypothesized reliability and validity thresholds were generally met.

**Conclusions:**

Proxy-SSPedi is reliable, valid and responsive in children 2-7 years old receiving cancer treatments. Mini-SSPedi can be used for children 6-7 years of age.

**Supplementary Information:**

The online version contains supplementary material available at 10.1186/s12885-022-09814-8.

## Background

The importance of active symptom screening and symptom monitoring for pediatric cancer patients has been increasingly recognized over time. Consequently, we developed and validated the Symptom Screening in Pediatrics Tool (SSPedi). SSPedi was designed for children and adolescents 8-18 years of age with cancer and pediatric hematopoietic stem cell transplant (HSCT) recipients. It asks respondents to self-report how much 15 symptoms bothered them yesterday or today on a 5-point Likert scale [1–3]. The proxy-report version of SSPedi was also validated for use in pediatric patients 8-18 years of age receiving cancer treatments [4].

An important gap was that SSPedi did not address the needs of younger children. For children 2-7 years of age, we reasoned that we could use the proxy-report version of SSPedi validated in pediatric patients 8-18 years of age but would need to confirm favorable psychometric properties. However, we took a different approach to create the self-report version of SSPedi for children younger than 8 years of age. We developed mini-SSPedi and focused on children 4-7 years because 4 is the age at which children are thought to be able to articulate concrete aspects about their health [5]. Mini-SSPedi was based upon SSPedi in that it includes the same 15 symptoms. However, it was modified as follows: focuses on “today” only rather than “yesterday or today”, uses a 3-point faces rather than a 5-point Likert scale and symptom descriptions were simplified. These modifications were based upon cognitive interviews with 100 children 4-7 years receiving cancer treatments [6]. The initial SSPedi development studies evaluated content validity for patients 8-18 years of age and their guardians [2]. While we did not reconfirm content validity among the younger cohort, we reasoned there were benefits in keeping the items the same and did not anticipate important differences in terms of content validation. Testing of the draft version of mini-SSPedi showed that it was understood and was not hard to complete [6].

With proxy and self-report versions of SSPedi for children 2-7 years and 4-7 years of age respectively now available, we were ready to evaluate the psychometric properties of these instruments. We hypothesized that proxy-SSPedi and mini-SSPedi would be reliable (test re-test reliability, inter-rater reliability and internal consistency) and valid (discriminate validity, convergent validity and responsive). Thus, objectives were to evaluate the reliability and validity of proxy-SSPedi and mini-SSPedi for pediatric patients receiving cancer treatments.

## Methods

This was a multi-center prospective observational study designed to evaluate the reliability, validity and responsiveness of proxy-SSPedi (2-7 years of age) and mini-SSPedi (4-7 years of age) in pediatric patients receiving cancer treatments or HSCT recipients.

### Subjects

Proxy respondents were guardians of pediatric patients 2-7 years of age with cancer or HSCT recipients. We excluded guardians who did not understand English and those with cognitive disability or visual impairment that precluded completion of proxy-SSPedi as determined by the child’s primary healthcare team. English-speaking children of participating guardians who were 4-7 years of age and whose illness severity, cognitive ability and visual status permitted completion of mini-SSPedi as determined by their primary healthcare team were eligible for optional participation in this study.

Two different participant groups were enrolled for the purpose of construct validation. One group was labelled the more symptomatic group and included eligible guardians of children and children themselves receiving active treatment for cancer or undergoing HSCT who were admitted to hospital or seen in clinic for four consecutive days. The second group was labelled the less symptomatic group and included guardians of children or children themselves with non-relapsed acute lymphoblastic leukemia who were at least 6 months into the maintenance phase of chemotherapy or those who had completed any cancer treatments at least 3 months prior to enrollment, who were clinically well and no procedures planned that day.

### Procedures

Respondents were recruited from London Health Sciences Centre (London, Ontario), The Hospital for Sick Children (Toronto, Ontario) and the Children’s Hospital of Eastern Ontario (Ottawa, Ontario). The Research Ethics Boards of The Hospital for Sick Children and all participating sites approved this study. Guardians provided informed consent and participating children provided assent for study participation. Potential respondents were identified in the inpatient or outpatient setting by a member of the study team. Participants in the more symptomatic group completed measures at enrollment and 3 days later (± 1 day) while participants in the less symptomatic group completed measures only at enrollment.

Demographic data were obtained from guardians and from the patient’s health records. At enrollment, guardians and participating children completed proxy-SSPedi and mini-SSPedi along with proxy and self-reported measures of symptoms or quality of life for the purpose of construct validation (Day 1). These measures were the Children’s International Mucositis Evaluation Scale (ChIMES), the Pediatric Nausea Assessment Tool (PeNAT), Faces Pain Scale-Revised (FPS-R) and a global quality of life (QoL) and an overall symptom visual categorical scale. For those in the more symptomatic group, the guardian and child (if applicable) completed proxy-SSPedi and mini-SSPedi a second time 3 days later along with a global symptom change scale (Day 4). Guardians reported whether symptoms overall were much worse, a little worse, the same, a little better or much better than the previous assessment (5-point scale) while children reported whether symptoms were worse, the same or better than the previous assessment (3-point scale).

### Instruments

Proxy-SSPedi consists of the following 15 symptoms: feeling disappointed or sad, feeling scared or worried, feeling cranky or angry, problems with thinking or remembering things, changes in how your body or face look, feeling tired, mouth sores, headache, hurt or pain (other than headache), tingly or numb hands or feet, throwing up or feeling like you may throw up, feeling more or less hungry than you usually do, changes in taste, constipation and diarrhea. Proxy respondents report their estimation of symptoms experienced by the pediatric patient. It uses a 5-point Likert scale (not at all bothered, a little, medium, a lot and extremely bothered) and it has a recall period of yesterday or today. Mini-SSPedi consists of the same 15 symptoms but with simplified descriptors. It uses a 3-point faces scale (not bothered at all, medium and extremely bothered) and it has a recall period of today. Proxy-SSPedi and mini-SSPedi were completed on paper for the first 188 guardians until the iPad version was available. The electronic version of mini-SSPedi reads the instrument and questions out loud as a default. A synonym list is available if children are having difficulty understanding the meaning of a symptom. If both guardian and child agreed to participate, the guardian completed proxy-SSPedi silently before the child completed mini-SSPedi. Guardians were instructed to not consult their child while they completed proxy-SSPedi. Next, participating children then completed mini-SSPedi without assistance from their guardian and without being able to see their guardian’s responses.

The other instruments were completed, by both guardians and children, on paper after completing proxy-SSPedi ± mini-SSPedi throughout the study. ChIMES is a reliable and valid measure of oral mucositis that is sensitive to change [7]. ChIMES results in two summary scores, which are the ChIMES Score (ranges from 0 to 23) and the Total ChIMES Percent (ranges from 0 to 100). For both summary scores, higher numbers indicate worse mucositis. PeNAT is a reliable and valid measure of present nausea severity, that ranges from 1 = “no nausea” to 4=“worst nausea possible”, in children four to 18 years of age [8]. The FPS-R is a reliable and valid measure of pain intensity in children four to 18 years of age that may be scored on a 0-10 scale. Higher numbers indicate more pain [9, 10]. Global QoL visual categorical scales are commonly used in research and are often used to validate other measures [11, 12]. We used a 5-point scale to assess global QoL ranging from 1 = “best possible” to 5 = “worst possible”. We also used a 4-point scale to assess overall symptoms ranging from 1 = “none” to 4 = “severe”. For both categorical scales, higher numbers indicate worse global QoL or overall symptoms.

At the completion of the interview, the research staff also adjudicated whether the child appeared to understand mini-SSPedi for participating children on a 4-point Likert scale ranging from 1 = “completely incorrect” to 4 = “completely correct”. The number of children who were partially correct (score of 3) or completely correct (score of 4) were tabulated.

### Statistics

For proxy-SSPedi, each item is scored as 0, 1, 2, 3 or 4 where the scores indicate 0=“not at all bothered”, 1 = “a little”, 2 = “medium”, 3 = “a lot” and 4 = “extremely bothered”. For mini-SSPedi, each item is scored as 0, 2 or 4 where the scores indicate 0 = “not at all bothered”, 2 = “medium” and 4 = “extremely bothered”. A total unweighted proxy-SSPedi or mini-SSPedi score was calculated for each administration where the scores for the 15 items were summed, resulting in a total score ranging from 0 (none) to 60 (worst possible).

Psychometric evaluation examined reliability, construct validity and responsiveness. The threshold criteria for reliability were derived from previously established recommendations [13]. To evaluate test-retest reliability of proxy-SSPedi and mini-SSPedi, we included those in the more symptomatic group who reported no change in symptoms between Day 1 and Day 4. We calculated the intraclass correlation coefficient (ICC) between the two proxy-SSPedi or mini-SSPedi total scores and we anticipated an ICC ≥ 0.75. To evaluate the inter-rater reliability of proxy-SSPedi and mini-SSPedi, we calculated the ICC between the baseline scores for dyads in which children were eligible and agreed to self-report. As in our previous SSPedi validation study, we anticipated an ICC ≥ 0.6 since guardians and children may have different perceptions of the child’s symptoms [14]. Finally, we evaluated internal consistency using the Cronbach’s alpha and anticipated an alpha > 0.8 [13].

In terms of construct validation, we evaluated discriminative or known-groups validity by hypothesizing that mean total proxy-SSPedi or mini-SSPedi scores would be significantly higher for participants in the more symptomatic group compared to the less symptomatic group. We compared the baseline total proxy-SSPedi and mini-SSPedi scores using the independent Student’s t-test. We also evaluated convergent validity by hypothesizing that there would be fair correlation (Spearman r ≥ 0.25) between the following measures: the mouth sores proxy-SSPed/mini-SSPedi item and Total ChIMES Percent; the nausea and vomiting proxy-SSPedi/mini-SSPedi item and PeNAT; the pain proxy-SSPedi/mini-SSPedi item and FPS-R; and total proxy-SSPedi/mini-SSPedi score and global QoL and overall symptom scales. The 95% confidence intervals around the Spearman r values were obtained through bootstrapping 1000 samples.

To evaluate the responsiveness of proxy-SSPedi and mini-SSPedi, the Day 1 and Day 4 scores for those in the more symptomatic group who reported symptoms to be much worse or much better for proxy-SSPedi (they completed a 5-point global symptom change scale), and worse or better for mini-SSPedi (they completed a 3-point global symptom change scale) were included. We used the paired Student’s t-test and accounted for difference in direction by multiplying the scores in the much better group by − 1.

The sample size calculation for test-retest reliability assumed the ICC under the null hypothesis was 0.5 and under the alternate hypothesis was 0.75, an α 0.05 and a β of 0.20. With these assumptions, we needed 36 guardians (two-tailed) who reported no change in symptoms between Day 1 and Day 4 [15, 16]. Assuming that 15-20% of guardians would provide a Day 4 assessment and would report no change in symptoms, 200 guardians in the more symptomatic group were targeted. For known groups validation, assuming a minimal clinically important difference of 5 points, standard deviation of 10, and α of 0.05, enrollment of 200 guardians in the more symptomatic group and 200 guardians in the less symptomatic group would provide > 99% power. Thus, the total targeted sample size was 200 guardians in the more symptomatic group and 200 guardians in the less symptomatic group (400 total). Given the anticipated number of children who would be eligible and agree to self-report, all mini-SSPedi analyses focused on description rather than hypothesis testing. Analyses were performed using R studio version 3.6.1, The R Foundation for Statistical Computing.

## Results

**Fig. 1:**
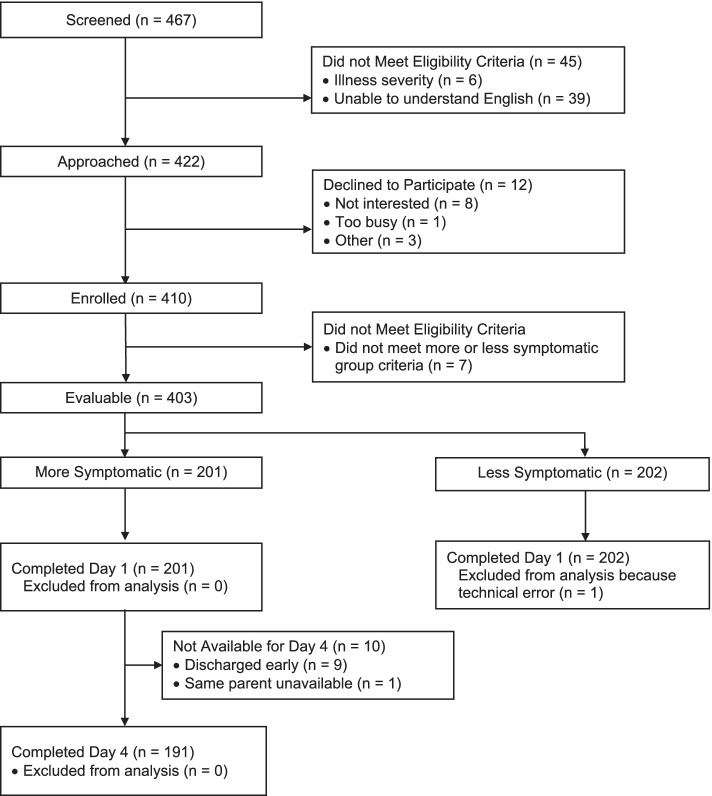
Flow Diagram of Participant Identification, Enrollment and Study Participation

Between February 1, 2018 and January 11, 2022, 467 guardians were assessed for eligibility. Among these, 402 guardians and 326 of their children were included in the analysis; 201 guardians and 159 children participated in the more symptomatic group, and 201 guardians and 167 children participated in the less symptomatic group. Figure 1 illustrates the flow diagram of participant identification, enrollment and study participation, and the reasons for exclusion.

Among the more symptomatic group guardians, a Day 4 assessment within the pre-specified window (±1 day) was obtained in 191/201 (95.0%) while among more symptomatic group children, a Day 4 assessment within the pre-specified window was obtained in 142/159 (89.3%). All enrolled participants completed proxy-SSPedi or mini-SSPedi and had no difficulty with completion. Among the 325 children who completed mini-SSPedi and in whom research staff adjudication of understanding was performed, 312 (88.6%) were partially or completely correct in understanding how to complete mini-SSPedi.

Table 1 shows the demographic characteristics of the study participants stratified by the more symptomatic and less symptomatic groups. Among the less symptomatic group, 56 were children with acute lymphoblastic leukemia in maintenance and 145 were cancer survivors. Overall, the median age of all child participants was 5.5 (range 2.0 to 7.9) years. The most common underlying cancer diagnosis was leukemia in 224 (55.7%) and 90 (22.4%) had metastatic disease. The most common guardian type was mothers in 290 (72.1%).

**Table 1:** Participant Demographics

Characteristic	Total No. (%)	More Symptomatic No. (%)	Less Symptomatic No. (%)
**Child Characteristics**	*N* = 402	*n =* 201	*n =* 201
Male	220 (54.7%)	116 (57.7%)	104 (51.7%)
Age in years			
2-3	57 (14.2%)	31 (15.4%)	26 (12.9%)
4-5	180 (44.8%)	108 (53.7%)	72 (35.8%)
6-7	165 (41.0%)	62 (30.8%)	103 (51.2%)
White	212 (52.7%)	96 (47.8%)	116 (57.7%)
Diagnosis			
Leukemia	224 (55.7%)	96 (47.8%)	128 (63.7%)
Lymphoma	13 (3.2%)	12 (6.0%)	1 (0.5%)
Solid tumor	118 (29.4%)	49 (24.4%)	69 (34.3%)
Brain tumor	23 (5.7%)	20 (10.0%)	3 (1.5%)
Other	24 (5.9%)	24 (11.9%)	0 (0.0%)
Metastatic disease	90 (22.4%)	52 (25.9%)	38 (18.9%)
Relapse	32 (8.0%)	30 (14.9%)	2 (1.0%)
Active treatment	238 (59.2%)	193 (96.0%)	45 (22.4%)
Treatments received			
Chemotherapy	382 (95.0%)	190 (94.5%)	192 (95.5%)
Surgery	117 (29.1%)	54 (26.9%)	63 (31.3%)
Radiotherapy	53 (13.2%)	30 (14.9%)	23 (11.4%)
Stem cell transplantation	44 (10.9%)	32 (15.9%)	12 (6.0%)
Inpatient at interview	193 (48.0%)	193 (96.0%)	0 (0.0%)
Reason for visit			
Chemotherapy or transplant	168 (41.8%)	148 (73.6%)	20 (10.0%)
Fever	26 (6.5%)	26 (12.9%)	0 (0.0%)
In school	284 (70.6%)	111 (55.2%)	173 (86.1%)
English as first language	340 (84.6%)	169 (84.1%)	171 (85.1%)
**Parent Characteristics**			
Male	106 (26.4%)	57 (28.4%)	49 (24.4%)
Relationship to patient			
Father	103 (25.6%)	56 (27.9%)	47 (23.4%)
Mother	290 (72.1%)	141 (70.1%)	149 (74.1%)
Other	9 (2.2%)	4 (2.0%)	5 (2.5%)
Married	343 (85.3%)	167 (83.1%)	176 (87.6%)
College or university education	328 (81.6%)	164 (81.6%)	164 (81.6%)
English as first language	268 (66.7%)	128 (63.7%)	140 (69.7%)
House income > $60,000	248 (61.7%)	123 (61.2%)	125 (62.2%)

Table 2 provides details of SSPedi administration. The median proxy-SSPedi Day 1 scores in the more and less symptomatic groups were 14 and 3 respectively. Median time to complete SSPedi was 2 minutes or less for all proxy respondents in both groups. The median mini-SSPedi Day 1 scores in the more and less symptomatic groups were 10 and 4 respectively. The median time to complete mini-SSPedi was about 5 minutes for both groups. Among the more symptomatic group, the global symptom change scale on Day 4 was reported as the same (no change in symptoms) in 45 (23.7%), and much better or worse in 47 (24.6%) among guardians, and the same in 28 (20.9%), and better or worse in 106 (79.1%) among children.

**Table 2:** Characteristics of Symptoms and Quality of Life Scores

	More Symptomatic (*n =* 201)		Less Symptomatic (*n =* 201)	
Outcome measures	Parent Proxy Report (*n =* 201)	Child self-report (*n =* 159)	Parent Proxy Report (*n =* 201)	Child self-report (*n =* 167)
Proxy-SSPedi or Mini-SSPedi				
Median total SSPedi scores day 1 (IQR)	14 (8 to 20)	10 (4 to 18)	3 (1 to 7)	4 (1 to 8)
Mean total SSPedi scores day 1 (SD)	14.8 ± 8.5	11.3 ± 9.8	5.1 ± 5.7	5.4 ± 5.9
Median minutes completion day 1 (IQR)*	2.0 (1.5 to 3.3)	5.1 (3.8 to 6.5)	1.6 (1.2 to 2.6)	5.0 (4.2 to 6.2)
Day 4 sample size	(*n =* 191)	(*n =* 142)	NA	NA
Median total SSPedi scores day 4 (IQR)	13 (7 to 18)	6 (2 to 12)		
Mean total SSPedi scores day 4 (SD) (*n =* 191)	13.2 ± 8.2	8.2 ± 8.7		
Median minutes completion day 4 (IQR)*	1.5 (1.1 to 2.2)	3.8 (3.2 to 4.9)		
Children’s International Mucositis Evaluation Scale				
Median ChIMES Scores (IQR)	0 (0 to 2)	0 (0 to 2)	0 (0 to 0)	0 (0 to 0)
Median ChIMES Percent (IQR)	0 (0 to 8.7)	0 (0 to 8.7)	0 (0 to 0)	0 (0 to 0)
Pediatric Nausea Assessment Tool, n (%)				
Nausea now				
No nausea at all	132 (66.3%)	132 (84.1%)	191 (95.0%)	153 (91.6%)
A little bit nauseated	41 (20.6%)	17 (10.8%)	8 (4.0%)	13 (7.8%)
Even more nauseated	16 (8.0%)	4 (2.5%)	2 (1.0%)	1 (0.6%)
Nauseated a whole lot	10 (5.0%)	4 (2.5%)	0 (0.0%)	0 (0.0%)
Vomited yesterday or today	63 (31.5%)	47 (29.7%)	6 (3.0%)	4 (2.4%)
Faces Pain Scale-Revised, median rating (IQR)	0 (0 to 3.5)	0 (0 to 2)	0 (0 to 0)	0 (0 to 0)
Global Quality of Life Categorical Scale, median (IQR)	3 (2 to 3)	1 (1 to 2)	1 (1 to 2)	1 (1 to 1)
Overall Symptom Scale, median (IQR)	2 (2 to 3)	1 (1 to 2)	1 (1 to 1)	1 (1 to 1)
Symptom change rating on day 4, No. (%)			NA	NA
Much worse	34 (17.9%)	90 (67.2%)		
A little worse	60 (31.6%)			
The same	45 (23.7%)	28 (20.9%)		
A little better	38 (20.0%)	16 (11.9%)		
Much better	13 (6.8%)			

* Available in *n =* 215 parents and *n =* 194 children who completed day 1 and *n =* 107 parents and *n =* 98 children who completed day 4 on an iPad

*Abbreviations*: *IQR* Interquartile range, *NA* Not applicable, *SD* Standard deviation

Table 3 summarizes the psychometric evaluation results. For test-retest reliability, the ICC for proxy-SSPedi and mini-SSPedi were 0.83 (95% CI 0.72-0.90) and 0.85 (95% CI 0.71-0.92) respectively and thus, met the a priori established threshold of ICC ≥ 0.75. In terms of inter-rater reliability, among the entire cohort, the ICC was 0.54 (95% CI 0.45-0.62) and consequently, it did not meet the a priori established threshold of ICC ≥ 0.6. However, when only children 6 and 7 years of age were included, the ICC was 0.62 (95% CI 0.50-0.71), which did meet the established threshold. In terms of internal consistency, total proxy-SSPedi was ≥0.8 for Day 1 and Day 4 evaluations.

**Table 3:** Psychometric Properties of Proxy-SSPedi and Mini-SSPedi

		Proxy-SSPedi		Mini-SSPedi	
Property	Hypothesis	No.	Results	No.	Results
Reliability					
Test-retest reliability	ICC ≥ 0.75 when comparing total SSPedi scores between days 1 and 4 in those who report no change in symptoms	45	ICC = 0.83, 95% CI 0.72 to 0.90	28	ICC = 0.85 95% CI 0.71 to 0.92
Inter-rater reliability	ICC ≥ 0.6 when comparing total SSPedi scores between children and parents on day 1	324	ICC = 0.54, 95% CI 0.45 to 0.62	NA	
Inter-rater reliability	ICC ≥ 0.6 when comparing total SSPedi scores between children and parents on day 1 for children 6 and 7 years	157	ICC = 0.62, 95% CI 0.50 to 0.71	NA	
Internal consistency	Total proxy-SSPedi and mini-SSPedi scores - Cronbach’s alpha ≥0.8				
	Day 1	402	alpha = 0.86, 95% CI 0.84 to 0.88	326	alpha = 0.79, 95% CI 0.75 to 0.81
	Day 4	191	alpha = 0.81, 95% CI 0.77 to 0.85	142	alpha = 0.79, 95% CI 0.72 to 0.84
Construct validity					
Known groups validity	Total SSPedi score higher for more symptomatic vs less symptomatic groups	402	Mean diff 9.7, 95% CI 8.3 to 11.1 *P <* 0.0001	326	Mean diff 5.9 95% CI 4.1 to 7.7 *P <* 0.0001
Convergent validity	Mouth soreness SSPedi item fairly correlated with Total ChIMES Percent, *r* = ≥0.25-0.50	402	Spearman *r =* 0.60 95% CI 0.50 to 0.68	326	Spearman *r =* 0.53 95% CI 0.42 to 0.64
Convergent validity	Nausea and vomiting SSPedi item fairly correlated with PeNAT, *r =* ≥0.25-0.50	402	Spearman *r =* 0.55 95% CI 0.45 to 0.64	326	Spearman *r =* 0.45 95% CI 0.30 to 0.59
Convergent validity	Pain SSPedi item fairly correlated with FPS-R, *r* = ≥0.25-0.50	402	Spearman *r =* 0.60 95% CI 0.52 to 0.67	326	Spearman *r =* 0.50 95% CI 0.37 to 0.62
Convergent validity	Total SSPedi score fairly correlated with global QoL scale, *r =* ≥0.25-0.50	402	Spearman *r =* 0.66 95% CI 0.59 to 0.72	326	Spearman *r =* 0.31 95% CI 0.20 to 0.41
Convergent validity	Total SSPedi score fairly correlated with overall symptom scale, r = ≥0.25-0.50	402	Spearman *r =* 0.74 95% CI 0.70 to 0.78	326	Spearman *r =* 0.39 95% CI 0.29 to 0.48
Responsiveness	Change in total SSPedi scores for the Much Worse or Much Better on day 4 vs day 1	47	Mean diff 7.7, 95% CI 5.6 to 9.9 *P <* 0.0001	106	Mean diff 3.1 95% CI 1.5 to 4.7 *P =* 0.0002

*Statistical tests to calculate two-sided *P* values were independent Student’s t test for known groups construct validity, and paired Student’s t test for responsiveness

*Abbreviations*: *CI* Confidence interval, *FPS-R* Faces Pain Scale – Revised, *ICC* Intraclass correlation coefficient, *PeNAT* Pediatric Nausea Assessment Tool, *QoL* Quality of life, *SSPedi* Symptom Screening in Pediatrics Tool, *diff* Difference

For known groups construct validation, the mean difference in total proxy-SSPedi scores between the more symptomatic and less symptomatic groups was 9.7 (95% CI 8.3-11.1, *P* < 0.0001) while the mean difference in total mini-SSPedi scores was 5.9 (95% CI 4.1-7.7, P < 0.0001). For convergent validity, all hypothesized relationships for both proxy-SSPedi and mini-SSPedi were observed. For responsiveness, the mean difference between Day 1 and Day 4 proxy-SSPedi scores for those who said their child was much better or much worse was 7.7 (95% CI 5.6-9.9, *P* < 0.0001) while the mean difference in mini-SSPedi scores for those who reported they were better or worse was 3.1 (95% CI 1.5-4.7, *P =* 0.0002).

## Discussion

We found that proxy-SSPedi (2-7 years of age) and mini-SSPedi (4-7 years of age) for children with cancer and pediatric HSCT recipients exhibited test re-test reliability, internal consistency, known groups validity, convergent validity and responsiveness. However, interrater reliability was established only for children 6 and 7 years of age. These results suggest that proxy-SSPedi may be used for clinical and research purposes in patients 2-7 years of age and mini-SSPedi may be used for children who are 6 and 7 years of age. Self-report symptom assessment may be less reliable in children younger than 6 years old.

While we found that children were able to complete mini-SSPedi, it required about 5 minutes to complete. The length of the instrument is likely related to the default audio administration since young children are unlikely to be able to read independently. It is reasonable that mini-SSPedi can be used for single or limited administrations for specific purposes, more likely in a research context. The length of time required may preclude frequent administration such as daily or several times per week but may be feasible for less frequent administration such as once weekly. Clinical implementation of more frequent symptom screening such as daily or multiple times per week may require a different approach and we have developed a formalized approach to the dyadic administration of SSPedi called co-SSPedi [14, 17].

We found that among the entire group, inter-rater reliability failed to meet our a priori established ICC threshold of ≥0.6. We used this same threshold for the validation of SSPedi for children and adolescents 8-18 years of age in which total self-report SSPedi scores were compared to guardian proxy-report scores [18]. However, a key difference between the two studies is that in the evaluation of SSPedi, the actual instruments completed by patients and guardians were essentially identical. However, proxy-SSPedi and mini-SSPedi are systematically different in several ways that are likely to impact on inter-rater reliability. First, the recall periods are different with the recall period of proxy-SSPedi being yesterday or today, and the recall period of mini-SSPedi being today. Second, the possible scores are different with proxy-SSPedi using a 5-point Likert scale and mini-SSPedi using a 3-point faces scale. Third, the description of symptoms is slightly different, with simpler descriptions in mini-SSPedi. Consequently, the threshold of 0.6 applied in this study was likely too high in the design of this study [19], suggesting that mini-SSPedi may be appropriate for all children 4-7 years of age.

Obtaining self-reported QoL outcomes for younger children is more challenging than for older pediatric patients [20]. Representation abilities develop at around age 3–5 years, with the ability of introspection about one’s own thoughts developing at age 6–8 years [5]. Children younger than 8 years of age may have difficulty in determining differences between the past, present and future [5], and this issue may have contributed to our low inter-rater reliability scores. Felder-Puig et al. reported on the validation of a QoL instrument among German-speaking children and included 29 children aged 5 to 7 years. Among these children, 11 were unable to provide self-report [21]. Children either refused to participate or did not understand the questions. Addressing self-reported QoL assessments in younger children has previously been identified as an important priority [20]. We believe a dyadic child-guardian approach may be promising and will be exploring it in future research [14, 17].

The strengths of this study are the multi-center approach and the inclusion of a wide variety of patient diagnoses and treatments. These elements improve the generalizability of the findings. However, the study is limited as it was only conducted in the English language. In addition, the study did not address the feasibility of repeated administrations that may be particularly challenging for very young children. Finally, ChIMES and the global QoL scale had not previously been validated among children 4-7 years of age. However, our findings support mutual concurrent validity of both instruments and mini-SSPedi.

In summary, proxy-SSPedi is reliable, valid and responsive in children 2-7 years old receiving cancer treatments. Mini-SSPedi can be used for children 6-7 years of age.

## Supplementary Information

**Additional file 1: Appendix 1.** Guardian-reported proxy-SSPedi scores among the more symptomatic group (*N =* 201)

## Data Availability

All data generated and analyzed is available on reasonable request.
