# Supplementary material for: Reliability and validity of proxy-SSPedi and mini-SSPedi in pediatric patients 2-7 years receiving cancer treatments
Source: BMC Cancer. 2022 Jul 4;22:730. doi: 10.1186/s12885-022-09814-8 (PMC9254487; doi:10.1186/s12885-022-09814-8)
Supplement: Supplementary file 1 — Additional file 1: Appendix 1. Guardian-reported proxy-SSPedi scores among the more symptomatic group (N = 201) [file 12885_2022_9814_MOESM1_ESM.docx]

**Appendix 1: Guardian-reported proxy-SSPedi scores among the more symptomatic group (N=201)**

|  | **Severely vs. Not Severely Bothersome Symptom** | **n (%)** |
| --- | --- | --- |
| Feeling disappointed or sad | not severe | 160 (79.6%) |
|  | severe | 41 (20.4%) |
| Feeling scared or worried | not severe | 161 (80.1%) |
|  | severe | 40 (19.9%) |
| Feeling cranky or angry | not severe | 150 (74.6%) |
|  | severe | 51 (25.4%) |
| Problems with thinking or remembering things | not severe | 198 (98.5%) |
|  | severe | 3 (1.5%) |
| Changes in how your body or face look | not severe | 179 (89.1%) |
|  | severe | 22 (10.9%) |
| Feeling tired | not severe | 134 (66.7%) |
|  | severe | 67 (33.3%) |
| Mouth sores | not severe | 186 (92.5%) |
|  | severe | 15 (7.5%) |
| Headache | not severe | 194 (96.5%) |
|  | severe | 7 (3.5%) |
| Hurt or pain (other than headache) | not severe | 169 (84.1%) |
|  | severe | 32 (15.9%) |
| Tingly or numb hands or feet | not severe | 195 (97.0%) |
|  | severe | 6 (3.0%) |
| Throwing up or feeling like you may throw up | not severe | 174 (86.6%) |
|  | severe | 27 (13.4%) |
| Feeling more or less hungry than you usually do | not severe | 150 (74.6%) |
|  | severe | 51 (25.4%) |
| Changes in taste | not severe | 174 (86.6%) |
|  | severe | 27 (13.4%) |
| Constipation (hard to poop) | not severe | 174 (86.6%) |
|  | severe | 27 (13.4%) |
| Diarrhea (watery, runny poop) | not severe | 179 (89.1%) |
|  | severe | 22 (10.9%) |
